# Syntaxin 12 Sustains Endosomal‐Lysosomal Homeostasis and Survival in Glioblastoma Stem‐Like Cells

**DOI:** 10.1111/tra.70042

**Published:** 2026-07-07

**Authors:** Clément Maghe, Agnieszka Barbach, Gwennan André‐Grégoire, Clotilde C. N. Renaud, Margaux Le Guyon, Rosalie Moreau, Nicolas Bidère, Julie Gavard

**Affiliations:** ^1^ CRCI^2^NA, Inserm, CNRS, Université de Nantes Nantes France; ^2^ Equipe Labellisée Ligue Contre le Cancer Paris France; ^3^ Integrated Center for Oncology, ICO Nantes France

**Keywords:** cell death, endo‐lysosome, glioblastoma, lysosomes, mTOR signaling, recycling, trafficking

## Abstract

Glioblastoma cells display a striking vulnerability to disruptions in late endosome‐lysosome trafficking, a dependency that we exploited through a targeted siRNA screen in patient‐derived cells with stem‐like properties (GSCs). This screen identified Syntaxin 12 (STX12) as a critical determinant of GSC survival. Originally characterized as a recycling endosome t‐SNARE, STX12 regulates tubular recycling and retrograde transport, yet its broader implications for lysosomal homeostasis remain poorly understood. Functional characterization revealed that STX12 is required to maintain lysosomal organization in GSCs, consistent with its known roles as an endosomal t‐SNARE. Loss of STX12 altered dynamic processes that support GSC fitness, culminating in controlling life‐and‐death decisions. Because lysosomal function is deeply connected with the autophagic pathway, we next explored whether STX12 contributes to this adaptive program. STX12 silencing produced signatures consistent with impaired endo‐lysosomal progression and disrupted mechanistic target of rapamycin (mTOR)/lysosome communication. This work further identifies a previously unrecognized role for STX12 in the adaptive trafficking network that supports glioblastoma cell survival, revealing a potential SNARE‐centered vulnerability for therapeutic intervention.

## Graphical abstract

Syntaxin12 maintains endolysosomal trafficking and lysosomal homeostasis in glioblastoma cells. Syntaxin12 depletion disrupts lysosomal organization, impairs endolysosomal progression and mTOR‐lysosome communication, and compromises adaptive pathways required for cell survival.

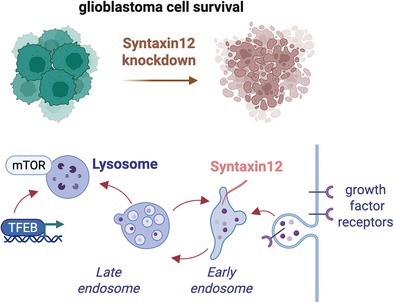

## 1 Introduction

Glioblastoma (GBM) is the most aggressive primary brain tumor in adults, with a median survival of approximately 15 months despite maximal surgical resection, followed by radiotherapy and temozolomide‐based chemotherapy, established by Stupp and colleagues in 2005 [1, 2]. A major contributor to therapeutic failure is the profound intra‐tumoral heterogeneity of GBM, which includes a subpopulation of tumor‐propagating cells commonly referred to as glioblastoma stem‐like cells (GSCs) [3]. These cells display enhanced self‐renewal capacity, resistance to genotoxic stress, and the ability to invade surrounding brain parenchyma, thereby driving recurrence and poor clinical outcome [4, 5, 6, 7]. Although the precise molecular definition and cellular origin of GSCs remain debated, their functional contribution to GBM maintenance and progression is well supported.

GSCs reside in specialized niches and continuously adapt to fluctuating and often hostile microenvironmental conditions, including hypoxia, nutrient limitation, and metabolic stresses [8]. To cope with these constraints, GSCs rely on highly plastic signaling and programs. Increasing evidence suggests that membrane trafficking pathways, particularly the endocytic and endo‐lysosomal systems, play a central role in these adaptive responses [9, 10, 11, 12]. Endocytic trafficking governs the spatio‐temporal control of receptor signaling, nutrient uptake, and metabolic homeostasis [13], while lysosomes function as hubs for macromolecule degradation, lipid metabolism, and signaling integration [14]. In keeping with this idea, alterations in lysosomal functions are increasingly recognized as hallmarks of cancer cells, including GBM [15, 16]. Cancer‐associated lysosomes often exhibit modified size, lipid and protein content, membrane properties, protease activities, and subcellular positioning, a palette of changes that can promote tumor expansion [15, 16]. In GSCs, lysosomal activity has been reported to be actively restrained, an adaptation thought to preserve stemness and survival under nutrient‐poor conditions [10, 12, 17]. Conversely, excessive or dysregulated lysosomal activation can engage non‐classical cell death pathways, such as lysosomal membrane permeabilization, highlighting lysosomes as potential therapeutic vulnerabilities [9, 10, 12].

Despite the recognized importance of the endo‐lysosomal compartment in cancer biology, the specific endomembrane trafficking regulators that GSCs depend upon for survival remain poorly defined. In particular, it is unclear whether GSCs exhibit selective dependencies on individual components of the endosomal machinery that could represent non‐oncogene addictions, that is genes that are not oncogenic per se but become essential for the survival of the transformed state. Identifying such dependencies could uncover tumor‐selective vulnerabilities that spare normal brain cells.

In this study, we performed an unbiased siRNA screen targeting membrane trafficking regulators to identify genes required for GSC survival. We identify STX12 as a tumor‐selective dependency in patient‐derived GSCs. Mechanistically, we show that STX12 loss disrupts early endocytic trafficking, alters plasma membrane receptor handling, and triggers a maladaptive lysosomal response characterized by transcription factor EB (TFEB) activation and increased lysosomal flux. Importantly, this response culminates in GSC cell death through a non‐canonical mechanism independent of classical apoptosis or necroptosis. Our findings uncover STX12 as a critical regulator of endosomal‐lysosomal homeostasis in GSCs and highlight endocytic trafficking as an actionable vulnerability in glioblastoma.

## 2 Results

### 2.1 An siRNA Screen Identifies STX12 as a Selective Vulnerability in Glioblastoma Stem‐Like Cells

**FIGURE 1:**
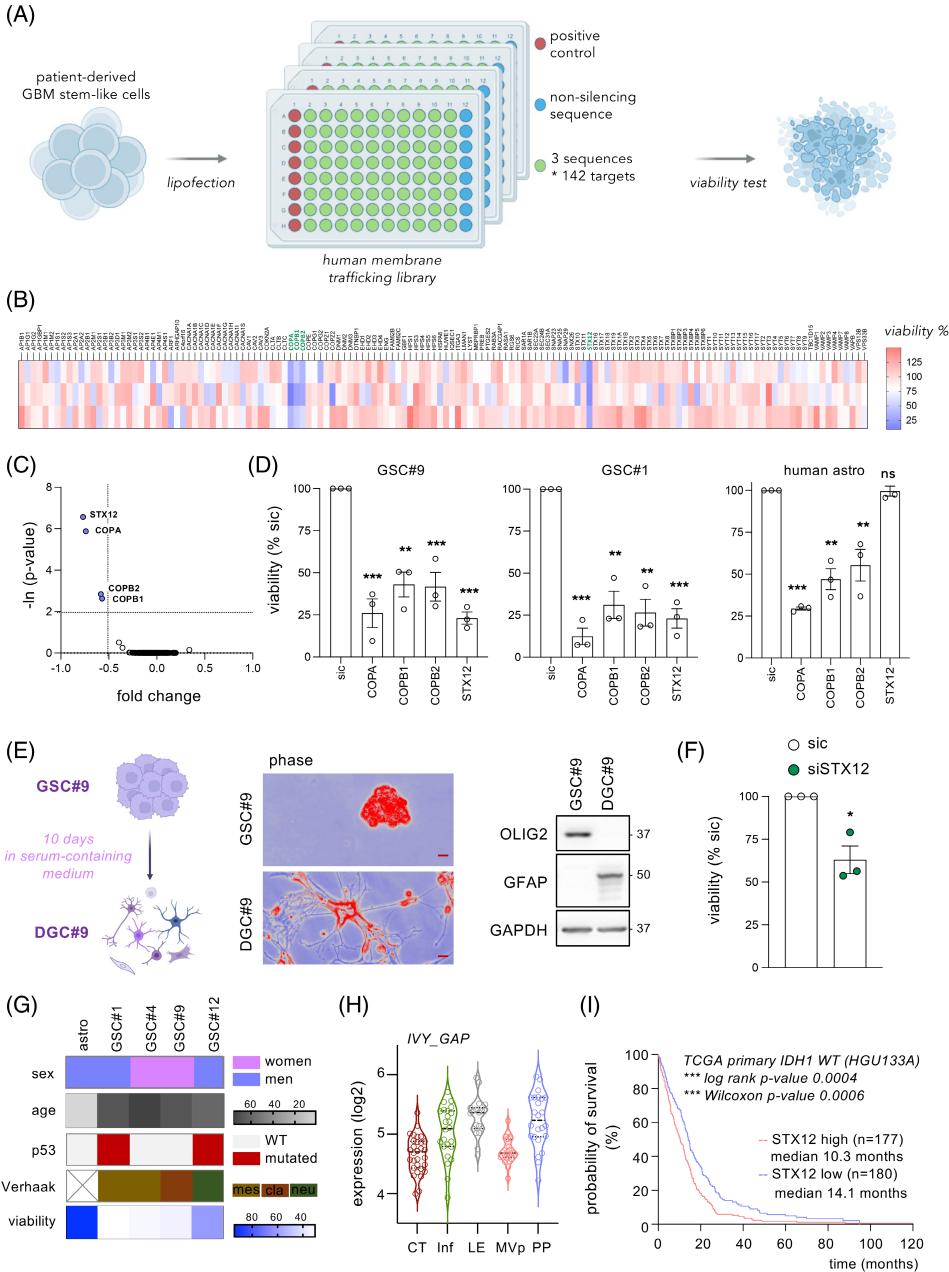
An siRNA viability screen identifies STX12 as a selective dependency in glioblastoma stem‐like cells. (A) Schematic of the siRNA‐based viability screen performed in patient‐derived GSC#9. A total of 142 trafficking‐related genes were targeted using three independent siRNAs per gene, with eight positive and eight negative controls per plate. (B) Heatmap showing normalized cell viability for each gene across the three siRNA sequences. Data were normalized to control duplexes (sic) transfected cells. (C) Volcano plot of mean viability fold change versus statistical significance (−ln *p*‐value) calculated across the three siRNAs per gene (cutoffs: Fold change ≤ −0.5; ln *p*‐value ≥ 2). Components of the COPI complex (COPA, COPB1, COPB2) and STX12 are highlighted. Data were normalized to control duplexes (sic) transfected cells. (D) Validation of candidate hits on cell viability in GSC#1, GSC#9, and primary human astrocytes. (E) Cartoon depicting the process to differentiate Glioblastoma Stem‐like Cells (GSC) into Differentiated Glioblastoma Cells (DGC). Representative phase‐contrast images showing the morphology of sphere‐forming patient‐derived GSC#9 and their differentiated counterpart DGC#9, after 10 days‐culture in differentiated medium. Scale bars: 10 μm. Western‐blot analysis of SOX2 and OLIG2, as stem and differentiation markers, respectively in GSC#9 and DGC#9. GAPDH serves as loading controls. Molecular weight markers are shown on the left. (F) Cell viability in sic‐ or siSTX12‐transfected differentiated glioblastoma cells DGC#9 after 96 h. (G) Heatmap summarizing viability fold change following STX12 knockdown in four independent GSCs, with distinct clinical and molecular features (sex, age, TP53 status, Verhaak subtypes). Human astrocytes serve as controls. (H) Analysis of STX12 expression in the Ivy Glioblastoma Atlas Project (IVYGAP) dataset showing enrichment in invasive tumor regions (Inf infiltrative zone, LE leading edge, PP pseudo‐palisading) relative to the tumor core (TC) and microvascular niche (MVp). (I) Kaplan–Meier survival analysis of TCGA glioblastoma patients stratified by STX12 expression, showing poorer overall survival in tumors with high STX12 levels. Each experiment was independently triplicated, and analyzed with ANOVA. ns, non‐significant, **p < 0.01, ***p < 0.001.

To systematically identify intracellular trafficking regulators required for GSC survival, we performed an siRNA‐based viability screen targeting 142 genes involved in endomembrane organization and vesicular transport and fusion (Figure 1A). Each gene was targeted with three independent siRNAs, enabling robust assessment of on‐target effects (Figure 1B). Using stringent cutoffs (fold change ≤ −0.5; −ln (*p*‐value) ≥ 2), this screen identified several candidates whose depletion significantly reduced GSC viability, including multiple COPI complex components (COPA, COPB1, COPB2) and the endosomal SNARE Syntaxin 12 (Figure 1C). SNARE proteins are central regulators of vesicular fusion events throughout the endomembrane system [18], yet their contribution to GSC survival and lysosomal homeostasis remains incompletely explored. Syntaxin 12 (STX12), also known as STX13, is an endosomal SNARE implicated in early and recycling endosome dynamics [18, 19, 20], but its function and relevance in GBM or stem‐like tumor cells has not been addressed.

Validation experiments revealed that COPI complex depletion impaired viability in both GSCs and primary human astrocytes, consistent with their essential cellular functions (Figure 1D) [21]. In contrast, STX12 knockdown selectively reduced viability in GSC1 and GSC9 while sparing healthy astrocytes, indicating a potential tumor‐selective dependency. To next address whether the role of STX12 is tumor‐selective or associated with stemness, we performed cell viability assays in differentiated glioblastoma cells (Figure 1E). These experiments show that differentiated GBM cells are moderately affected by STX12 knockdown, in contrast to glioblastoma stem‐like cells (Figure 1F). This suggests that STX12 dependency might be enriched in the stem‐like state rather than being a general vulnerability across all GBM cell populations. However, while this differential sensitivity may be linked to the differentiation status itself, we cannot exclude that the observed resistance of differentiated cells is also influenced by culture conditions, including their adherent growth state and the use of serum‐containing medium.

This selective requirement for STX12 was confirmed across four independent patient‐derived GSC lines representing diverse clinical and molecular backgrounds (Figure 1G), including sex, age, TP53 status, and Verhaak subtypes. Analysis of the IVY Glioblastoma Atlas Project (IVYGAP) dataset showed that STX12 expression is enriched in infiltrative and invasive GBM tumor regions, including the infiltrative zone, leading edge, and pseudo‐palisading zones, indicative of a potential role in tumor invasion (Figure 1H). Moreover, high *STX12* RNA expression was associated with worse overall survival in TCGA glioblastoma patients (Figure 1I), supporting a potential clinically relevant role for STX12 in this aggressive disease.

### 2.2 STX12 Depletion Triggers GSC Cell Death Independently of Canonical Apoptosis or Necroptosis

**FIGURE 2:**
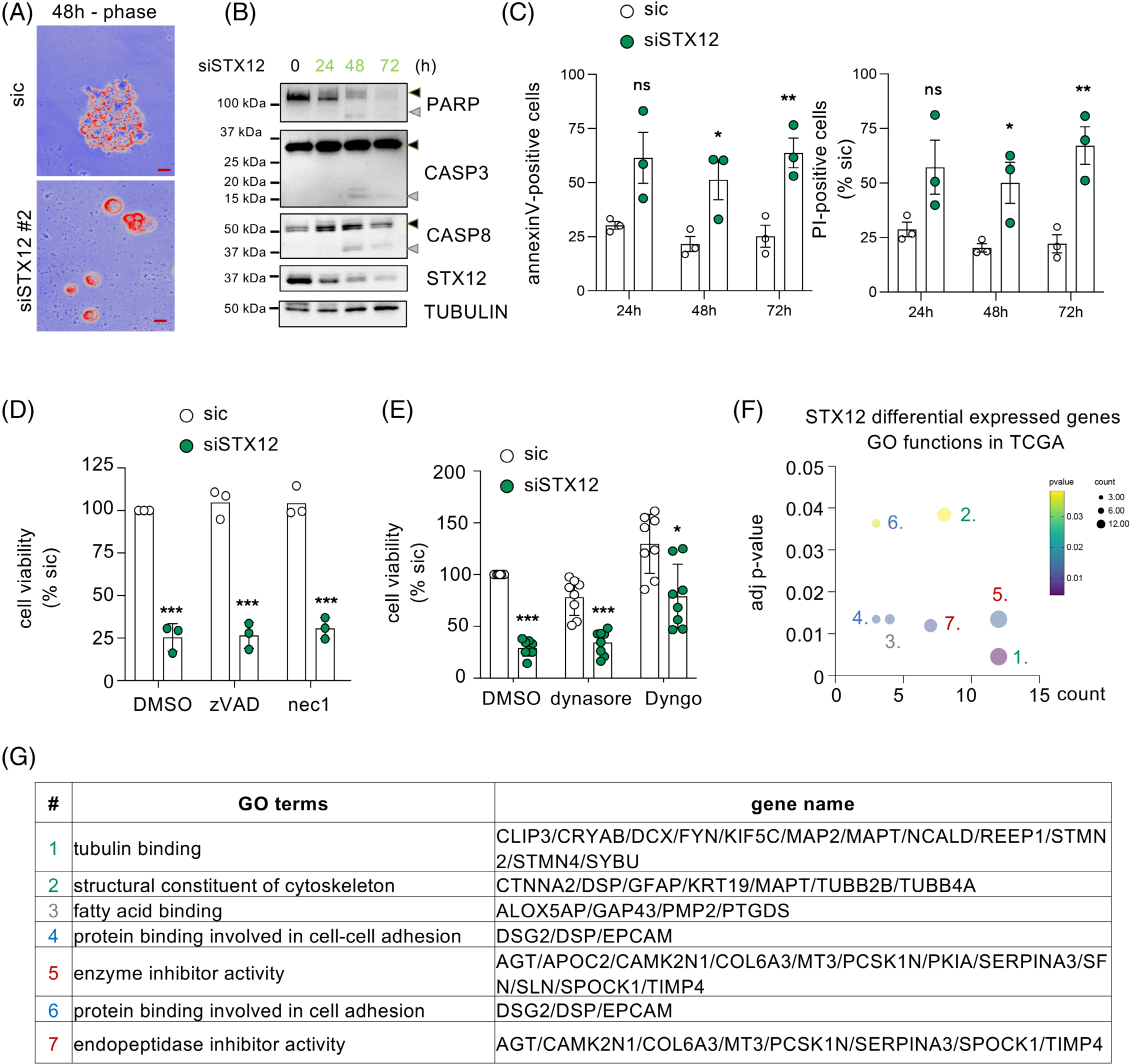
STX12 knockdown induces non‐canonical cell death in GSCs. (A) Representative phase‐contrast images of tumorspheres formed by GSCs transfected with control duplexes (sic) or STX12 siRNA (siSTX12) after 48 h. Scale bars: 10 μm. (B) Western‐blot analysis showing time‐dependent cleavage of CASP3, CASP8, and PARP following STX12 knockdown. TUBULIN serves as a loading control. Molecular weight markers are shown on the left. (C) Flow cytometric analysis of Annexin V and propidium iodide (PI) staining in siC or siSTX12‐transfected cells. (D) Cell viability following treatment with the pan‐CASP inhibitor zVAD‐fmk zVAD, 20 μM or the necroptosis inhibitor necrostatin‐1 (nec1, 20 μM) in sic‐ or siSTX12‐transfected cells after 48 h. (E) Cell viability following inhibition of endocytosis with Dyngo‐4a (10 μM) or dynasore (40 μM), in sic or siSTX12‐transfected cells. (F, G) Gene ontology (GO) analysis of TCGA datasets identifying biological processes associated with STX12‐correlated transcripts. Each GO‐term and associated genes are listed. Each experiment was independently triplicated and analyzed with ANOVA. ns, non‐significant, *p < 0.05, **p < 0.01, ***p < 0.001.

STX12 knockdown markedly impaired in vitro GSC growth, as evidenced by reduced tumorsphere formation (Figure 2A). At the molecular level, STX12 depletion induced time‐dependent cleavage of caspase‐3 (CASP3), CASP8, and PARP (Figure 2B), accompanied by increased Annexin‐V and propidium iodide (PI) staining (Figure 2C), indicating activation of cell death pathways.

Despite CASP activation, their inhibition with zVAD‐fmk failed to restore cell viability. Similarly, targeting necroptosis with necrostatin‐1 had no overt impact (Figure 2D), suggesting that STX12 loss induces a non‐canonical or mixed cell death program. Of interest, inhibition of dynamin‐dependent endocytosis with Dyngo‐4a partially rescued viability, whereas the less potent first‐generation inhibitor dynasore [22] had no effect (Figure 2E), implicating an alteration of endocytic trafficking as a contributor to the cell death observed. Finally, gene ontology analysis of TCGA datasets further linked STX12 expression to pathways involved in tubulin and cytoskeletal organization, lipid metabolism, and enzymatic activity, consistent with a global role in endosomal and metabolic regulation (Figure 2F,G).

### 2.3 Loss of STX12 Disrupts Early Endocytic Trafficking and Receptor Handling

**FIGURE 3:**
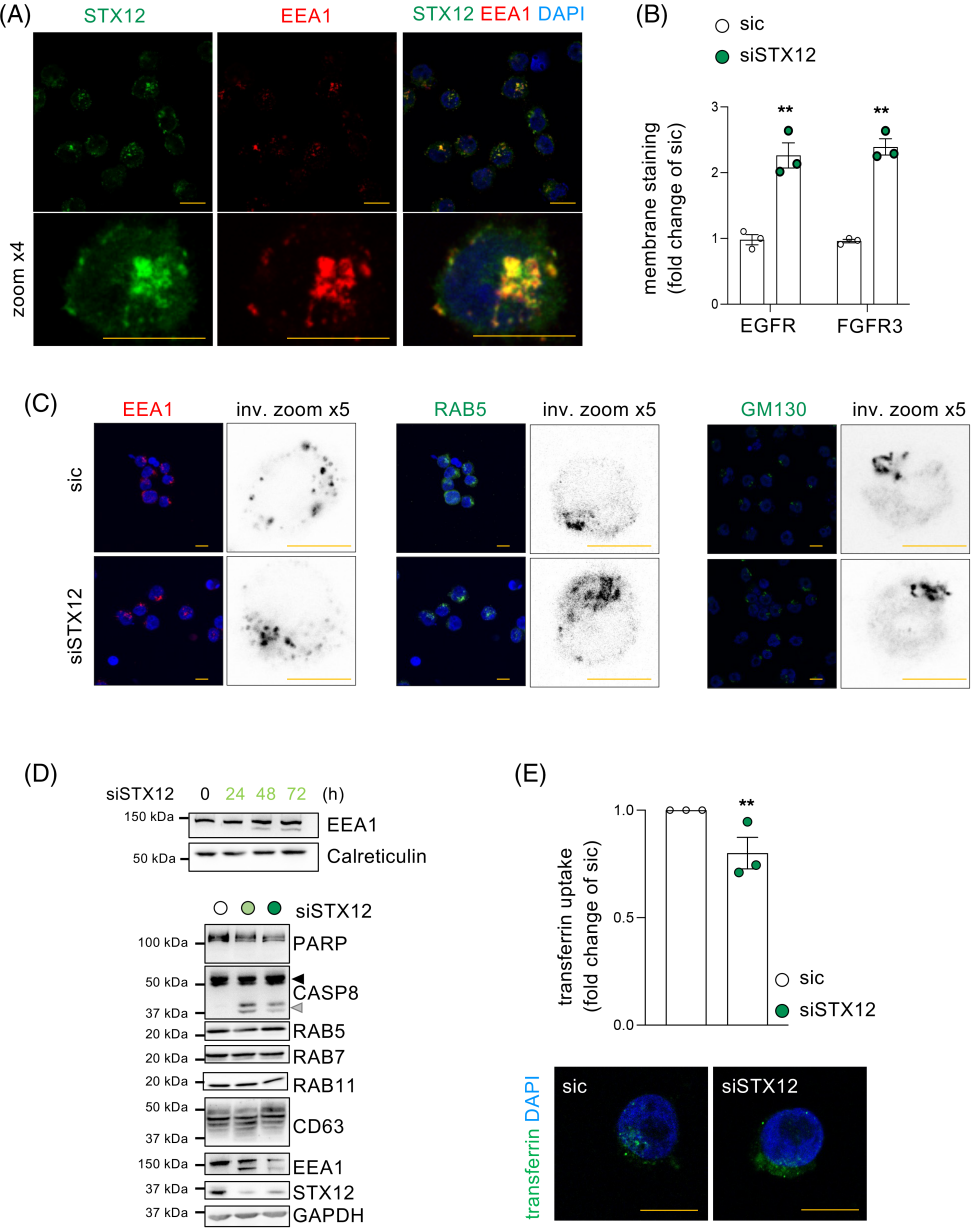
STX12 depletion disrupts early endocytic trafficking in GSCs. (A) Confocal immunofluorescence images showing colocalization of STX12 (green) with the early endosome marker EEA1 (red) in GSCs. Nuclear staining with DAPI is shown in blue. Corresponding higher‐magnification insets are shown below. Scale bars: 10 μm. (B) Flow cytometric analysis showing increased surface accumulation of EGFR and FGFR3 in GSCs transfected with control duplexes (sic) or STX12 siRNA (siSTX12). (C) Confocal imaging of EEA1 (red), RAB5 (green), and GM130 (green) in control and STX12‐depleted cells, in sic or siSTX12‐transfected cells. Nuclear staining with DAPI is shown in blue. Corresponding higher‐magnification insets with inverted grayscale are shown on the right. Scale bars: 10 μm. (D) Western‐blot time course using two independent STX12 siRNAs showing progressive cleavage of EEA1 coincident with CASP8 and PARP activation and STX12 knockdown, without changes in total CD63, RAB5, RAB7, or RAB11 levels. CALRETICULIN and GAPDH serve as loading controls. Molecular weight markers are shown on the left. (E) Quantification of transferrin uptake (green) assay analyzed by confocal microscopy in sic or siSTX12‐transfected cells. Scale bars: 10 μm. Each experiment was independently triplicated and analyzed with ANOVA. ns: non‐significant, **p < 0.01.

In line with earlier characterization in HeLa cells, STX12 colocalized with the early endosome marker EEA1 in GSCs (Figure 3A). Furthermore, STX12 knockdown resulted in increased surface accumulation of EGFR and FGFR3 (Figure 3B), suggesting impaired receptor internalization.

While the Golgi organization appeared largely preserved as seen with GM130 staining, STX12 depletion led to a modest, increased accumulation of EEA1 and RAB5‐positive vesicles (Figure 3C). However, western‐blot analysis revealed no changes in total levels of RAB5, RAB7, RAB11, and CD63 following STX12 knockdown (Figure 3D). However, a potent cleavage of EEA1 was noted, in line with EEA1 being previously reported as a substrate of CASP3 and CASP1 in the course of programmed cell death [23, 24]. Functionally, and in line with the accumulation of the surface receptors EGFR and FGFR3, transferrin uptake was moderately but consistently reduced (Figure 3E). These findings suggest that STX12 depletion does not affect the overall abundance of major endosomal markers, but rather impacts endosomal organization and trafficking dynamics.

### 2.4 STX12 Depletion Enhances Lysosomal Flux Through TFEB Activation

**FIGURE 4:**
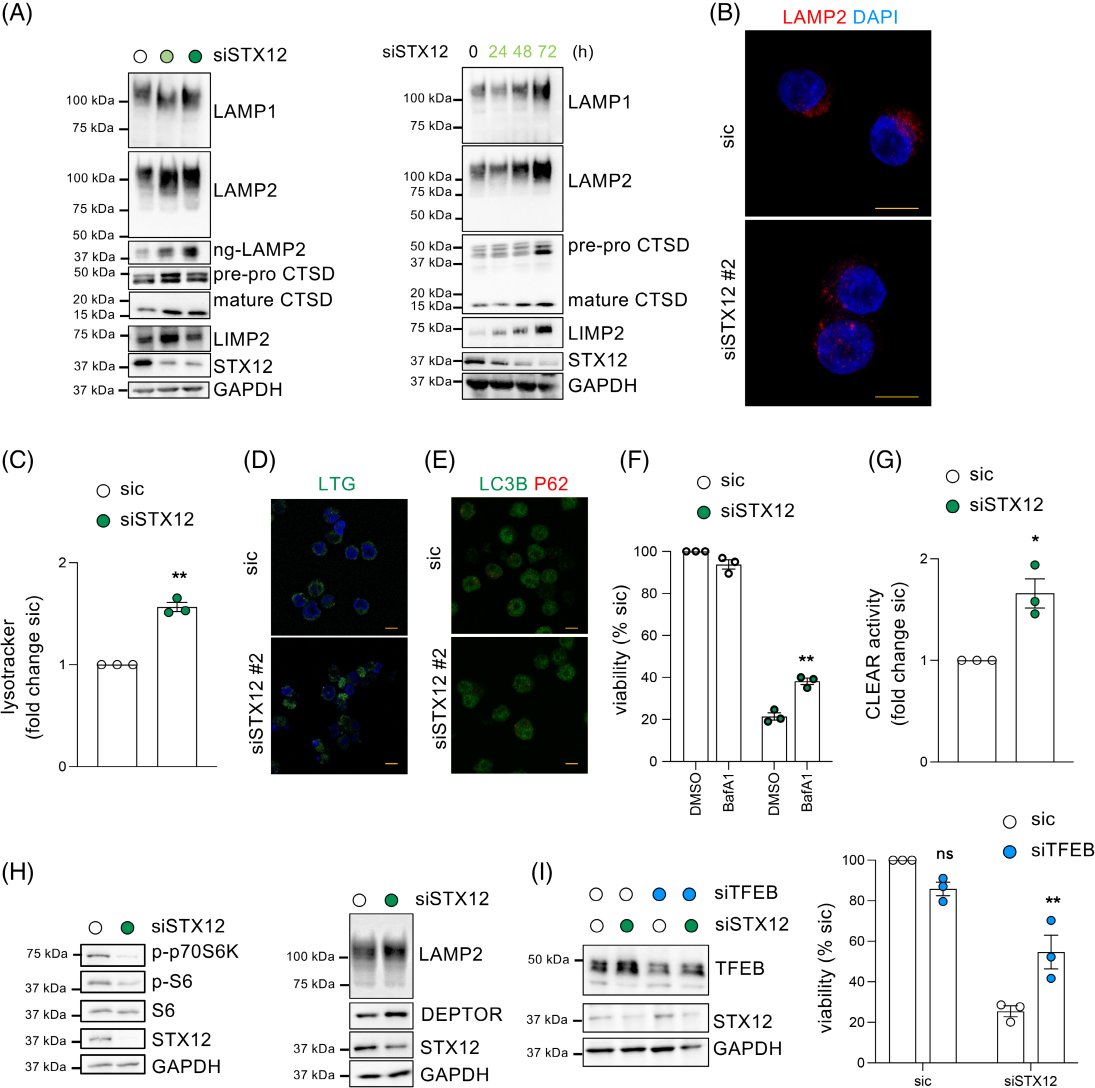
STX12 knockdown enhances lysosomal flux through TFEB activation. (A) Western‐blot analysis showing increased expression of late endosome/lysosome markers (LAMP1, LAMP2, non‐glycosylated LAMP2, Cathepsin D, LIMP2) following STX12 depletion, assessed over time and with two independent siRNAs. GAPDH serves as a loading control. Molecular weight markers are shown on the left. (B) Confocal images showing LAMP2 staining (red) in sic and siSTX12‐treated GSCs. Nuclear staining with DAPI is shown in blue. Scale bars: 10 μm. (C, D) Flow cytometry quantification and representative confocal images of LysoTracker staining (green), in sic or siSTX12‐transfected cells. Nuclear staining with DAPI is shown in blue. Scale bars: 10 μm. (E) Confocal analysis of LC3 puncta (green) and P62 (red) showing no significant induction of autophagosome formation. Scale bars: 10 μm. (F) Cell viability following inhibition of autophagic flux with Bafilomycin A1 (100 nM) endocytosis in sic or siSTX12‐transfected cells. (G) CLEAR network luciferase reporter assay in sic or siSTX12‐transfected cells. (H) Western‐blot analysis showing reduced mTOR signaling activity, as indicated by decreased phosphorylation of p70S6K and S6 and increased DEPTOR levels. GAPDH serves as a loading control. Molecular weight markers are shown on the left. (I) Validation of TFEB knockdown by western‐blot and corresponding viability assays in sic or siSTX12‐transfected cells. Each experiment was independently triplicated and analyzed with ANOVA. ns: Non‐significant, *p < 0.05, **p < 0.01.

As the early endocytic machinery seemed to be altered upon loss of STX12, we next interrogated lysosomal features. STX12 knockdown induced a robust increase in late endosome and lysosome‐associated luminal and membrane‐anchored proteins, including LAMP1, LAMP2 (including non‐glycosylated forms), Cathepsin D (including pre‐pro and mature/active processed forms), and LIMP2, as shown by western‐blot analysis using two independent siRNAs over time (Figure 4A). These changes were accompanied by a moderate increase in intensity and altered distribution of LAMP2 staining (Figure 4B), alongside an elevated LysoTracker staining measured by both flow cytometry and confocal microscopy (Figure 4C,D), indicating increased lysosomal mass and/or acidity.

Despite the marked alteration in the lysosomal compartment, STX12 depletion did not induce autophagosome formation, as LC3 puncta were not visible (Figure 4E). Likewise, Bafilomycin A1 treatment that inhibits autophagic flux did not exacerbate STX12‐driven cell death (Figure 4F). Instead, it resulted in a modest but reproducible increase in cell viability. Because STX12 depletion may engage in a maladaptive or pro‐death response, potentially reflecting cellular stress linked to endolysosomal dysfunction, we investigated the potential for TFEB/mechanistic target of rapamycin (mTOR) signaling. STX12 knockdown significantly increased the transcriptional activity of the CLEAR (Coordinated Lysosomal Expression and Regulation) network, indicating activation of its master gene TFEB [25] (Figure 4G). Consistent with this, mTOR Complex 1 (mTORC1) signaling was suppressed, as evidenced by reduced phosphorylation of p70S6K and S6. Of interest, the protein level of the mTORC1 negative regulator DEPTOR was increased (Figure 4H).

Importantly, TFEB knockdown efficiently reduced TFEB protein levels and significantly rescued the loss of viability induced by STX12 depletion (Figure 4I), demonstrating that aberrant TFEB‐dependent lysosomal activation is a key mediator of STX12‐dependent GSC death.

## 3 Discussion

Syntaxin 12 (STX12, also referred to as STX13) was originally identified as an endosome‐localized member of the syntaxin family with roles in recycling endosome dynamics and retrograde trafficking between endosomes, the Golgi, and the plasma membrane. Early studies established STX12 as a t‐SNARE involved in tubular endosomal extensions and cargo recycling, with additional links to constitutive and regulated exocytosis [18, 19, 20]. Despite this foundational work, the physiological consequences of disrupting STX12‐dependent trafficking, particularly in the context of stress‐adapted cells, such as glioblastoma stem‐like cells (GSCs), have remained poorly understood.

Our data identify STX12 as a non‐oncogene addiction gene in patient‐derived GSCs, and to a milder extent in differentiated glioblastoma cells (DGCs), as we revealed that its loss triggers a maladaptive endocytic‐lysosomal response culminating in cell death. Importantly, the phenotypes observed upon STX12 depletion cannot be explained by a simple model in which increased surface abundance of growth factor receptors enhances survival signaling. Instead, the combination of impaired clathrin‐mediated endocytosis (CME), reduced transferrin uptake, accumulation of EGFR and FGFR at the plasma membrane, and progressive cell death is most consistent with a membrane‐ and mistrafficking‐induced death phenotype. Accordingly, our data show a moderate but consistent reduction in transferrin uptake upon STX12 knockdown, indicating that receptor‐mediated iron uptake is most likely affected. Given the observed alterations in endosomal trafficking upon STX12 knockdown, including perturbed early endosomal organization, it is plausible that perturbation of transferrin trafficking could in turn influence intracellular iron handling. Since ferroptosis is highly dependent on iron availability and tightly linked to lysosomal and endolysosomal function [12, 26], such alterations may contribute to GSC vulnerability under STX12 depletion. Overall, these findings suggest that ferroptosis‐related alterations may represent a putative downstream consequence of disrupted endosomal homeostasis upon STX12 depletion.

It is increasingly appreciated that productive EGFR and FGFR signaling requires receptor internalization and signaling from endosomal compartments, where spatial organization enables sustained activation of downstream pathways, such as PI3K‐AKT and ERK [27, 28]. In this context, our findings suggest that STX12 loss does not simply reduce endocytosis but disrupts the coordination and maturation of endocytic trafficking, thereby preventing efficient receptor internalization that may result in improper endosomal signaling outputs. The resulting collapse of harmonious, spatially organized signaling might contribute to the activation of intrinsic cell death pathways, consistent with our observation that cell death proceeds independently of classical apoptosis or necroptosis. The differential effects of Dyngo‐4a and dynasore help to clarify this model [22]. Importantly, Dyngo‐4a does not appear to rescue viability by further blocking endocytosis in an already impaired system. Instead, we propose that partial and selective dynamin inhibition may dampen aberrant clathrin‐coated pit dynamics and membrane stress induced by STX12 loss, thereby restoring a minimal but functional endocytic flux. In this scenario, Dyngo‐4a rebalances a dysregulated trafficking system rather than suppressing it outright, allowing, for instance, sufficient endosomal EGFR/FGFR signaling to be re‐established and enabling reactivation of key survival pathways. In contrast, dynasore fails to rescue viability, likely due to its broader effects on membrane cholesterol, actin dynamics, and mitochondrial function, further exacerbating membrane and metabolic stress, and therefore further compromising residual trafficking capacities. Together, these findings suggest that it is not the absolute reduction of endocytosis per se that drives cell death, but rather the qualitative disruption and imbalance of endocytic dynamics.

EEA1 is a canonical Rab5 effector that localizes to PI3P‐enriched early endosomes, where PI3P serves as a key identity lipid required for EEA1 membrane recruitment and retention [29]. Perturbation of early endosomal fusion through STX12 knockdown is therefore expected to destabilize Rab5‐positive/PI3P‐positive early endosomal membranes, leading to impaired EEA1 association and increased protein instability or turnover. In parallel, PI3P is also present on maturing endosomal compartments, including CD63‐positive multivesicular bodies, where it contributes to endosomal maturation and cargo sorting [30].

Beyond early endocytic defects, STX12 depletion induces a pronounced lysosomal response characterized by TFEB activation, expansion of lysosomal markers, and altered mTOR signaling. Prior studies have linked STX12 to autophagy and mTOR‐regulated stress responses, including roles in autophagosome maturation and neuronal injury responses [31, 32]. Our findings extend this framework by showing that, in GSCs, loss of STX12 does not necessarily induce canonical autophagy but instead drives excessive TFEB‐dependent lysosomal activation that decreases cell fitness. The rescue of cell viability upon TFEB knockdown underscores that this lysosomal response is maladaptive rather than protective.

Together, these results position STX12 as a critical organizer of an adaptive trafficking network that integrates CME, receptor signaling, and lysosomal homeostasis. In GSCs, which are under constant environmental and metabolic stress, disruption of this network appears sufficient to drift the balance from survival to death. More broadly, our study highlights how dependence on specific SNARE‐mediated trafficking steps can constitute a non‐oncogene addiction in cancer cells and suggests that targeting endo‐lysosomal coordination may expose selective vulnerabilities in glioblastoma.

## 4 Materials and Methods

### 4.1 Cell Culture and Inhibitors

All patient‐derived glioblastoma stem‐like cells (GSCs) were cultured according to the French Ministry of Higher Education and Research rules under the #DUO10524 authorization. GSCs were dissociated from primary glioblastoma tissue (MACS Dissociator, Miltenyi). All subjects have given their informed consent. This study was approved by the institutional review boards of Saint Anne Hospital (Paris, France) and Laennec Hospital (Nantes, France), and performed in accordance with the Declaration of Helsinki Protocol. They were characterized for their self‐renewal capabilities, expression of stemness markers, cell surface antigens, their ability to differentiate, and to initiate tumor formation. GSC#4 (mesenchymal, 76‐year‐old female), GSC#6 (mesenchymal, 68‐year‐old male), GSC#9 (classic, 68‐year‐old female), GSC#13 (proneural, 73‐year‐old female), and GSC#15 (mesenchymal, 72‐year‐old male) were grown as spheres in mitogen‐defined serum‐free medium (NS34 DMEM/F12 with G5, B27, and N2 supplements, GlutaMAX and antibiotics, Life Technologies [33]). Human fetal astrocytes SVGP12 were cultured as per the manufacturer's instructions. (CRL‐8621, ATCC). Differentiated glioblastoma cells (DGC9) were generated by culturing GSC9 in DMEM/F12 with 10% fetal bovine serum (FBS), Glutamax, and antibiotics (Life Technologies) for 10 days. The following drugs were all from Selleckchem and added 6 h after siRNA transfection and maintained over 72 h: bafilomycin A1 (100 nM), dynasore (40 μM), Dyngo‐4a (10 mM), zVAD.fmk (20 mM), and necrostatin‐1 (20 mM). DMSO served as a vehicle.

### 4.2 siRNA Transfection

RNA duplexes targeting the respective human genes were transfected in 0.5 × 10^6^ cells in 0.6 mL media, and at 133 nM final concentration using RNAiMAX Lipofectamine (Life Technologies), as per the manufacturer's instructions. Stealth non‐silencing low‐GC RNA duplexes were used as a non‐silencing control (low‐GC 12935111, Life Technologies) and siRNA targeting human MALT1 as a positive control (CCUGUGAAAUAGUACUGCACUUACA, Cat#10620312) [9]. *Silencer* Human Membrane Trafficking siRNA Library (142 genes, Life Technologies, Cat#A30139) was used for the screening and validation shown in Figure 1. Additional sequences targeting human STX12 (CCUAUGGAGACAGUAAUUATT, light green symbol, GGGCCCAAAUGCAUAAGUUTT, dark green symbol) and TFEB (AGACGAAGGUUCAACAUCATT) were purchased from Life Technologies.

### 4.3 Protein Cell Lysis and Western‐Blot

Cells were harvested on ice, washed in cold PBS, pelleted (500×g, 3 min, 4°C), and lysed in RIPA buffer (25 mM Tris–HCl pH 7.4, 150 mM NaCl, 0.1% SDS, 0.5% Na‐Deoxycholate, 1% NP‐40, 1 mM EDTA) supplemented with Halt protease inhibitor cocktail for 30 min on ice. Lysates were then cleared by centrifugation (10 000 *g*, 10 min, 4°C) to pellet insoluble debris and nuclei. Protein concentrations were determined using a micro‐BCA assay kit. 10 μg of post‐nuclei supernatants were resolved by SDS‐PAGE and transferred to nitrocellulose membranes. Proteins were fixed to the membranes using a Ponceau S solution, and nonspecific protein binding sites were saturated with 5% milk in PBS‐Tween 0.05%. Primary and secondary antibodies were incubated with membranes in a similar blocking solution. Signals were revealed using chemiluminescent HRP substrate and visualized using Fusion imaging system (Vilber‐Lourmat). The following primary antibodies were used at a 1:1000 dilution, unless otherwise stated: PARP (Santa Cruz Biotech SC‐8007), CASP3 (CST 9662), CASP8 (CST 9746), STX12 (Proteintech 14 259‐1‐AP), TUBULIN (Santa Cruz Biotech SC‐8035), EEA1 (BD Transduction 610 456), CALRETICULIN (CST 122238), RAB5 (CST 3541), RAB7 (CST 9367), RAB11A (Abcam ab65200), CD63 (SBI exo1B‐CD63‐A1), GAPDH (1:10.000 dilution, Santa Cruz Biotech SC‐47724), LAMP1 (Santa Cruz Biotech SC‐19992), LAMP2 (Santa Cruz Biotech SC‐18822), LIMP2 (Santa Cruz Biotech SC‐55570), CSTD (Santa Cruz Biotech SC‐374381), p‐p70S6K T421 (CST 9204), pS6 S235/236 (CST 2211), S6 (CST 2317), DEPTOR (Proteintech 20 985‐1‐AP), SOX2 (Merck Millipore AB5605), OLIG2 (Merck Millipore AB9610), and TFEB (CST 3778). HRP‐conjugated secondary antibodies were from Southern Biotech (1080‐05, 1090‐05, 1010‐05, 4050‐05, 1070‐05) at a 1:5000 dilution.

### 4.4 Immunofluorescence Staining

Cells were fixed with PBS‐PFA 4% for 10 min, permeabilized with PBS‐TritonX‐100 0.05% for 5 min, and saturated with PBS‐BSA 3% for 1 h. Immunofluorescence was performed using primary antibodies diluted in PBS‐BSA 3%. The following primary antibodies were used at a 1:100 dilution: STX12 (Proteintech 14 259‐1‐AP), EEA1 (BD Transduction 610 456), RAB5 (CST 3541), GM130 (Abcam ab52649), LC3B (CST 3868), and P62 (CST 88588). Lysosomes were stained with Lysotracker (1:400, L7528, Life Technologies). Following washes, Alexa‐conjugated secondary antibodies from Life Technologies (A‐21123, A‐21133, A‐21144, A‐21131, A‐11035, A‐21121) were used at a 1:1000 dilution.

Cells were then incubated with DAPI (1:1000, Sigma Aldrich) for 5 min, and mounted in a mounting medium (Prolong gold anti‐fade reagent, Life Technologies). Fluorescence was observed with NIS‐Element Software at the Nikon Excellence Centre (Micropicell, UMS Biocore, Nantes, France). Images were acquired using an oil‐immersion lens. All images were analyzed using the ImageJ software.

### 4.5 Cell Viability Assay

Cell viability was measured using a luminescent metabolic assay (CellTiter‐Glo, Promega) following the manufacturer's protocol. Experiments were performed in 96‐well plates in a final volume of 100 μL culture medium per well. GSCs were seeded at 8000 cells per well in triplicate for each condition and, when needed, further challenged with siRNA transfection. Viability was read after 4 days on the FluoStar Optima plate reader (BMG Biotech).

### 4.6 Transferrin Uptake and Flow Cytometry

Following treatment, cells were washed with culture medium and incubated with Alexa594‐conjugated transferrin (25 μg/mL, T13343) for 30 min at 37°C. Cells were subsequently acid‐washed for 40 s, fixed in 4% PFA for 10 min, and analyzed with either flow cytometry on FACSCalibur (BD Biosciences, Cytocell) with data processed in FlowJo software, or confocal microscopy with DAPI nuclei counterstaining (Nikon A1 Rsi, MicroPicell). Alternatively, staining for FITC‐conjugated FGFR3 (FAB7662F, R&D systems) and APC‐conjugated EGFR (FAB1129A, R&D systems) was performed on living cells (incubation, 1 h at 4°C), together with appropriate controls for gating (IC0041A and NBP1‐96849).

### 4.7 CLEAR Reporter Assay

The CLEAR network luciferase assay was performed by transfecting GSC#9 with 1 μg plasmid (Addgene #66800) using a Neon Transfection System. 24 h later, cells were transfected with the indicated siRNA as previously described and assayed 2 days later using the Dual‐Glo Luciferase assay system (Promega) according to the manufacturer's guidelines. Luminescence was measured on the FluoStarOptima plate reader.

### 4.8 Bioinformatics Analysis Using Public Datasets and Online Tools

The TCGA for glioblastoma (IDHwt) was interrogated through cBioportal (https://www.cbioportal.org/) and Gliovis (https://gliovis.bioinfo.cnio.es/), and the Ivy Glioblastoma Atlas Project resource was retrieved from IVY‐GAP (https://glioblastoma.alleninstitute.org/rnaseq/search/index.html).

### 4.9 Schematics

Schematic cartoons in panels were created with BioRender.com.

### 4.10 Statistics

Data are representative of at least three independent experiments, unless otherwise stated. Statistical analysis was performed with GraphPad Prism10 using *Student's t‐*test, one‐way and two‐way ANOVA, as specified.

## Author Contributions

C.M., A.B., G.A.‐G., C.C.N.R., M.L.G., R.M., and J.G. performed experiments and analyzed data; C.M., N.B., and J.G. designed the study; C.M. and J.G. prepared the figures; J.G. wrote the manuscript. All authors approved the version.

## Funding

This work was supported by Fondation ARC contre le Cancer (ARCPJA2021060003730), (ARCPJA2025080010397), Institut National Du Cancer INCa PLBIO (2023‐044, INCa PAIR‐CEREB lNCa_16285), Ligue Nationale Contre le Cancer (EL2022), and Comités Ligue 35, 44, 49, 72, 85, and Conseil Régional des Pays de la Loire, Trajectoire Nationale. CM received a fellowship from Ligue Contre le Cancer. The team is part of the SIRIC ILIAD (INCa‐DGOS‐Inserm_12558).

## Ethics Statement

The authors have nothing to report.

## Conflicts of Interest

The authors declare no conflicts of interest.

## Data Availability

This study includes no data deposited in external repositories. All relevant data can be found in the manuscript.
